# A COMMUNITY-DRIVEN INITIATIVE TO ENGAGE EMPLOYERS AS ADVOCATES TO SUPPORT WORKING CAREGIVERS

**DOI:** 10.1093/geroni/igad104.0553

**Published:** 2023-12-21

**Authors:** Kylie Meyer, Morgan Zachmeyer, Jane Paccione, Christina Smith, Cynthia Cardenas, Dianne Teran, Carol Zernial

**Affiliations:** Case Western Reserve University, Cleveland, Ohio, United States; WellMed Charitable Foundation, San Antonio, Texas, United States; San Antonio Area Foundation, San Antonio, Texas, United States; WellMed Charitable Foundation, San Antonio, Texas, United States; Good Samaritan Community Services, San Antonio, Texas, United States; Alzheimer’s Association, San Antonio, Texas, United States; WellMed Charitable Foundation, San Antonio, Texas, United States

## Abstract

Approximately 62% of family caregivers to older adults living with a chronic illness or disability are employed. Nearly 6 in 10 caregivers report that caregiving has affected their employment in the last year, such as by requiring them to turn down a promotion, reduce work hours, or leave the workforce. Loss of employment income affects caregivers’ immediate and long-term financial well-being. To help family caregivers balance employment and caregiving and promote their long-term financial security, in 2021, the Successfully Living and Aging in San Antonio Caregiver/Socialization Workgroup developed a 5-part discussion series to encourage local employers to consider how to better support employees who are caregivers. The series was attended by 75 individuals representing 34 organizations. During initial discussions, attendees identified the importance of institutional culture in affecting workplace policies around caregiving. To help promote supportive workplace cultures, in the third discussion session, participants used a modified nominal group technique to co-develop a 10-point pledge that employers can use support employees who are caregivers. In this presentation, the authors will describe the development of the pledge. Further, we contextualize this initiative in terms of the Shilton Model of Policy Advocacy. By integrating employers into discussions about supporting employees who are caregivers, this local initiative provides an example of how communities can incorporate employers as stakeholder advocates to support policies to help family caregivers remain in the workplace. This work is particularly valuable to advance policy recommendations to promote caregiver financial well-being within the 2022 National Strategy to Support Family Caregivers.

